# Young Child Nutrition: Knowledge and Surveillance Gaps across the Spectrum of Feeding

**DOI:** 10.3390/nu14153093

**Published:** 2022-07-28

**Authors:** Elizabeth J. Reverri, Mary Beth Arensberg, Robert D. Murray, Kirk W. Kerr, Karyn L. Wulf

**Affiliations:** 1 Abbott Nutrition, Abbott Laboratories, Columbus, OH 43219, USA; mary.arensberg@abbott.com (M.B.A.); kirk.kerr@abbott.com (K.W.K.); karyn.wulf@abbott.com (K.L.W.); 2 Department of Pediatrics, The Ohio State University, Columbus, OH 43219, USA; murraymd@live.com

**Keywords:** nutrition guidelines, young child nutrition, toddler nutrition, young child formula, nutrient gaps

## Abstract

The first 1000 days is a critical window to optimize nutrition. Young children, particularly 12–24 month-olds, are an understudied population. Young children have unique nutrient needs and reach important developmental milestones when those needs are met. Intriguingly, there are differences in the dietary patterns and recommendations for young children in the US vs. globally, notably for breastfeeding practices, nutrient and food guidelines, and young child formulas (YCFs)/toddler drinks. This perspective paper compares these differences in young child nutrition and identifies both knowledge gaps and surveillance gaps to be filled. Parental perceptions, feeding challenges, and nutrition challenges are also discussed. Ultimately, collaboration among academia and clinicians, the private sector, and the government will help close young child nutrition gaps in both the US and globally.

## 1. Introduction

The first 1000 days are a critical window to optimize nutrition. This time period includes young children 12–24 months of age [1]. During this second year of life, the dietary patterns of young children transition to predominantly table foods. Young children have unique nutrient needs and begin to reach important developmental milestones to support nutrition: motor skills to self-feed, language skills to communicate with caregivers, and social customs acculturation [2]. Young child nutrition has both short- and long-term consequences on health, especially brain development. In particular, the adequacy of key macronutrients and micronutrients is important [1].

Adequate nutrition during the first 1000 days can have a striking impact on economic outcomes in adulthood too. Fortifying young children’s diets during this vulnerable phase has been found to increase their likelihood of completing secondary and college-level education and of early adult employment [3]. Other studies have shown adequate young childhood nutrition to increase reading comprehension, cognitive ability, and wages in adulthood [4]. The long-term benefits of successful young childhood nutrition can make it a highly cost-effective intervention.

Despite the cost-effectiveness of nutrition, guidelines for and practices related to young child nutrition, including intake of breast milk, food, and young child formulas (YCFs)/toddler drinks, vary substantially across countries. Additionally, the ability of caregivers to implement nutrition recommendations can vary across and within countries. Our perspective paper compares young child nutrition in the US and globally and based on this suggests knowledge and surveillance gaps across the spectrum of young child feeding. A food-based approach is discussed; dietary supplements are not addressed but have been recently reviewed by others [5].

## 2. Breastfeeding Practices

Breast milk is considered the gold standard nutrition for the first year of life. The American Academy of Pediatrics recommends continuing breastfeeding, along with appropriate complementary foods at about six months through the first two years of life or beyond, as long as mutually desired by mother and child [6]. However, during the second year of life, only 7–12% of young children in the US still receive human milk, consuming about 320 mL/day [7,8] or 20% of a toddler’s energy needs. This percent of energy needs is in contrast to the sole source of nutrition of breast milk and/or infant formula during infancy [9]. While inadequate supply and pain are common reasons why women may stop breastfeeding during infancy [10], less is known about the discontinuation of breastfeeding in young children.

The World Health Organization (WHO) recommends continued breastfeeding for up to two years of age or beyond along with solid foods. The Global Breastfeeding Scorecard estimates that 70% of young children worldwide are breastfeeding at 12 months of age, which decreases to 45% at 24 months. Globally, breast milk provides one-third of energy needs between 12–24 months of age [11]. The few studies that have investigated why women stop breastfeeding early are usually country-specific. Shared factors across countries related to breastfeeding cessation include HIV positive status, more than four children, and employment [12].

*Knowledge and Surveillance Gaps*:

- There is an opportunity to expand on data collection for breastfeeding practices between 12–24 months and beyond to include increased frequency, nutritional composition, and reasons for breastfeeding cessation.
- Length of maternity leave varies worldwide. Comparison of maternal employment, duration of breastfeeding, and impact on young child nutrition is an important avenue of exploration.
- In addition, more prenatal and postnatal education and family support is needed to promote breastfeeding guidelines.

## 3. Nutrient and Food Guidelines

By about 12 months of age in the US, the largest change in dietary patterns has occurred as infants move into young childhood: breastfeeding or feeding infant formula has decreased, while table food consumption has increased [13]. The nutrient density of foods remains a critical focus because, compared to adults, young children’s requirements for macronutrients and micronutrients per kilogram of body weight are higher [14]. 

Many current nutrient and food guidelines for young childhood are either undergoing or recently underwent revision. The US National Academies of Sciences, Engineering, and Medicine Dietary Reference Intakes (DRIs) identify specific nutrient levels needed for different age groups, including for young children [9]. The US Food and Drug Administration (FDA) used information from the DRIs, National Health and Nutrition Examination Survey (NHANES), and US Dietary Guidelines for Americans (DGAs) to update the Daily Value percentages that are expressed on US Nutrition Facts food labels [15]. The DRIs are often cited in the international literature, although the Food and Agricultural Organization (FAO) of the United Nations and WHO have also developed macronutrient and micronutrient guidelines. These FAO-WHO global nutrient requirements for young children up to three years of age are in the process of being updated, starting with calcium, vitamin D, and zinc [11].

In addition to helping inform nutrition labeling, DRIs also provide a framework for analyzing the results of food consumption surveys. US surveys, such as the NHANES and Feeding Infants and Toddlers Study, have reported important nutrient gaps, including inadequate/low intakes of fiber, potassium, vitamin D, and vitamin E by toddlers [16,17]. Such data were considered when healthy dietary patterns for young children were included for the first time in the 2020–2025 US DGAs. For a young child in the second year of life, the US DGAs identified multiple dietary components of public health concern: underconsumption of vitamin D, calcium, fiber, and potassium and overconsumption of sodium, saturated fat, and added sugars [13]. Globally, although specific nutrient deficiencies vary by country, deficiencies in iodine, vitamin A, and iron are among the most important, particularly for young children [11].

Data on the actual diets of young children are often limited, particularly because their dietary patterns substantially change and often vary based on breastfeeding practices. Further, diet-based research tools may not be available. For example, dietary quality can be difficult to assess since the Healthy Eating Index starts at two years of age. The US DGAs summarized food group intake for toddlers ages 12–23 months and found that toddlers in the US mostly consumed every food group each day, although data was limited to percentages of food groups and a few subgroups [13]. Globally, it has been reported that one-fourth of young children 6–23 months of age meet dietary diversity and feeding frequency appropriate for age [11]. Yet data is still scarce. The Global Dietary Database project documented less than one-third of individual-level dietary intake surveys had dietary intake for 0 to <2-year-olds [18].

To help inform the required nutrients and their levels, food-based dietary guidelines have been developed. The US DGAs emphasize nutrient dense foods, a food group/subgroup pattern including vegetables, fruits, grains, dairy, protein foods, and oils, and responsive feeding for 12–23 month-olds. For 2- to 4-year-olds, the US DGAs also encourage nutrient-dense foods, in addition to physical activity and a food group/subgroup pattern encouraging variety and multiple exposures to foods [13]. The FAO maintains a global database of food-based dietary guidelines from >100 countries and the United Nations Children’s Fund (UNICEF) has a global database of feeding indicators, or standards of data collection, from >100 countries for young children ages 6–23 months old [19]. Across organizations, consistent recommendations for toddlers are a diverse diet, including a variety of nutrient-dense foods, to support growth and development.

Further, the US DGAs recommend the introduction of potentially allergenic foods along with other complementary foods [13]. Overall, food allergies have been increasing worldwide and are estimated to be 8% in children [20]. Less is known in developing countries, but data so far suggests that specific food allergies vary by geographic region [21]. In the US, food allergies increased 50% between 1997 and 2011, and 5.6 million children have a food allergy, with 40% of them having multiple food allergies [22]. Management of food allergies includes avoiding offending allergens and consuming suitable substitutes. However, micronutrient deficiencies and poor growth frequently occur [23]. The burden of food allergies ranges from lower quality of life, increased caregiver burden and greater anxiety, social consequences, and financial costs [24].

*Knowledge and Surveillance Gaps*:

- Given recent/ongoing revisions of guidelines, it will take time for healthcare professionals and caregivers to be educated on changes. As such, surveillance methods may need to be updated to reflect these changes. One consideration in the US is better alignment of US DGAs and DRIs: US DGAs separate the toddler years into two groups (12–23 months of age and 2–8 years of age) versus the DRIs for toddlers are based on 12–36 months of age.
- Although current surveillance studies do provide valuable insight into the nutritional status of young children, more dietary intake data are needed, particularly regarding at-risk nutrients.
- Currently, nutrient and food guidelines are available at the national level in many countries around the world. Identification of subpopulations will help to move guidelines closer toward more personalized nutrition.

## 4. Young Child Formula (YCF)/Toddler Drink Guidelines

Toddler drinks in the US and YCFs outside of the US are options to help fill nutritional gaps in young children’s diets. They have varying nutrient levels and serving sizes, and different names for this category are used globally. Codex Alimentarius (Codex) recently aligned with the term, drink for young children with added nutrients [25].

The US DGAs state that there is no clear need for toddler drinks, as nutrients may be obtained from other foods, and toddler drinks may contain added sugars [13]. In addition, a US consensus statement on healthy beverages does not recommend toddler drinks for healthy, young children, with the rationale being there is no unique nutritional value beyond what can be obtained by healthy foods [26]. A similar rationale has been considered internationally, such as by the European Food Safety Authority (EFSA), although their scientific opinion states that YCFs are one of several ways to increase inadequate/at risk of inadequate status of alpha-linolenic acid (ALA), docosahexaenoic acid (DHA), iron, vitamin D, and iodine in European young children [27]. WHO has a statement that YCFs are unnecessary and may negatively impact breastfeeding [11].

Yet on a practical basis, YCFs/toddler drinks may be clinically recommended and chosen by caregivers to specifically help meet young child nutrient needs and address feeding and growth challenges. One international expert group published compositional recommendations for YCFs worldwide. They leveraged dietary nutrient intake and biochemical status data from young children in 17 countries and compared it to WHO nutritional requirements, identifying several nutrients often with inadequate intake in young children’s diets: vitamins A, D, B12, and C, and folate, calcium, iron, iodine, and zinc [28]. The authors proposed that YCFs are one of several options when combined with an age-appropriate diet that can contribute to increasing nutrient consumption, thereby reducing micronutrient deficiencies [28]. Similarly, a European Society for Paediatric Gastroenterology, Hepatology, and Nutrition (ESPGHAN) position paper on YCF included a systematic review, determining that, while routine use of YCFs is not necessary, they can be one of several strategies to increase the nutrient intake of iron, vitamin D, and omega-3 polyunsaturated fatty acids (PUFAs), as well as decrease protein intake from unfortified cow’s milk in European young children [29]. Additional YCF compositional guidelines and expert statements have been published for specific populations outside of the US (Table 1).

There are also a growing number of published clinical studies showing value with beneficial short-term effects of YCFs (Table 2). These double-blind, randomized, controlled, clinical trials were of strong study designs, although there were a limited number and heterogeneity in populations, formulations, duration, serving sizes, and ages [35,36,37,38]. All but one clinical study demonstrated beneficial effects of primary outcomes that ranged from nutrient status with improved lipid profile, increased folate levels [36], and preserved iron status [35] to body composition with a lower percentage of body fat [37] to immune parameters with increased total blood immunoglobulin A level [38]. These findings are encouraging; however, more clinical studies are needed.

In the US, there are no defined nutrient requirements for toddler drinks [15]. Internationally, YCFs for 12–36 months of age are covered by a specific Codex Standard for Follow Up Formulas (under revision), which many countries adopt into their local legislation. This established standard for specific nutrient ranges facilitates the manufacturing, marketing, and trade of products specifically formulated for older infants and young children >6 months of age in Codex-compliant countries [25]. Combined, the expert guidelines/statements, clinical studies, and scientific standards provide guidance on the nutritional composition of YCFs, which can help to inform future product development and clinical studies.

One important area that is less well-studied is parental views on how they can best meet their young child’s nutritional needs. A recent online survey investigated the parental satisfaction and perceived benefits of toddler drinks of 300 parents in the US with a toddler with a mean age of 16 months. Half of the parents felt that their toddler was a picky eater. Parents rated overall nutritional adequacy as their nutritional reason for providing a toddler drink, and calcium, iron, and B vitamins were the most liked ingredients of toddler drinks [44]. Another online survey of 1186 parents in four Latin American countries reported on parental satisfaction/confidence level of their young children’s general health. Compared to parents who did not use YCFs, parents who served YCF felt more confident that their young children were getting adequate nutrition, such as protein, vitamins, and minerals (*p* < 0.01). In the survey, parents using YCF were providing at least one serving/day (defined as 180 mL accounting for added water and excluding powder displacement), although 50% of these parents were providing two servings/day [45].

*Knowledge and Surveillance Gaps*:

- There is a need to better standardize toddler drinks in the US. Both in the US and globally alignment on serving size, nutritional composition, and inclusion or exclusion of powder displacement would be helpful.
- Expert statements on YCFs for specific subpopulations are needed.
- Clinical studies and added surveillance will help with understanding specific young child subpopulations who may benefit most from YCFs, in conjunction with the promotion of a healthy diet.
- A knowledge gap is parental perceptions of young child nutrition and how to support parents in their young child feeding decisions.

## 5. Feeding and Nutrition Challenges during Young Childhood

Childhood dietary patterns are shaped by various factors, including feeding and nutrition challenges [13], and long-term outcomes are at stake. Birth weight triples in the first 12 months, and birth length doubles by the age of five years. Brain volume doubles in 12 months and triples by 36 months. The need for energy and nutrients to support growth necessitates the addition of solid foods to augment the use of breast milk and/or infant formula after the age of six months [46,47]. The increasing provision of table foods during the second year of life exposes young children to similar foods as caregivers and, along with the provision of more complex food combinations, collectively shape young children’s dietary patterns by 24 months [47]. Deficiencies or imbalances in dietary patterns may lead to malnutrition [11]. The highest risk for malnutrition occurs at weaning, around 12 months in the US, but often later in the second year globally. The quality of the diet becomes critical to support growth and development. Typically, dairy milk assumes much of the nutritional foundation that was previously provided by breast milk and/or infant formula [47,48].

Several feeding issues raise the risk for malnutrition, some individual to the young child, such as food neophobia, temperament, and intensity of response to bitter taste, and others related to family demographics, such as education, income, food insecurity, and cultural norms. Young children accept what is familiar and routine. Preferences are “trained” through familiarity. Food acceptance requires that caregivers show patience, persistence, and a willingness to repeatedly offer previously rejected foods. Studies suggest that 8 to >15 exposures may be needed [46].

Caregivers who label their young child as selective or “picky” commonly halt the presentation of rejected foods after only 3–5 tries. They also may attribute their young child’s limited food acceptance to inheritance, rather than learned behavior. Bribing or pressuring the child to eat, along with a permissive feeding style of catering to the child, fosters food rejection. It is normal for young children to display “food jags” (wanting the same food repeatedly) and to show shifting preferences. Although some young children may demonstrate an exaggerated phobia toward new foods, these are likely not permanent rejections.

Providing a diverse array of nutrient-rich foods at every meal and snack can help overcome these challenges and allows young children to sample and express preferences. The term, “responsive feeding,” describes a reciprocal relationship between the child and the caregiver at mealtime and is commonly recommended. Similar guidance is offered by the US DGAs and by the US Centers for Disease Control and Prevention [13,49].

There is no widely accepted definition of “picky” eating, and thus, globally, it appears there is limited consensus on appropriate assessment tools, and prevalence estimates vary considerably [50]. In addition, few studies have assessed the association of “picky” eating with pediatric development, although there are indications that such feeding patterns may have negative effects [51]. Continued breastfeeding into the second year or use of YCFs may offer dietary support as behavioral issues are addressed [46,47].

Other factors that impact feeding and nutrition challenges during young childhood are social determinants of health. The WHO defines social determinants of health as non-medical factors influencing health outcomes [11]. Healthy People 2030 organizes them into five domains: economic stability, education access and quality, health care access and quality, neighborhood and built environment, and social and community context [52]. Health inequalities exist throughout the world. In particular, food insecurity affected 14.8% of US households with children in 2020 and has been correlated with childhood obesity [53]. In the US, toddlers living in households with limited incomes may be supported by US federal government programs, such as Special Supplemental Nutrition Program for Women, Infants, and Children, and Supplemental Nutrition Assistance Program, among others [13].

Screen media time is another important nutrition challenge that especially affects the health of young children in developed countries. In fact, both the WHO and AAP discourage screen time for young children under the age of 18 months, with the AAP only recommending if introducing it to 18–24-month-olds, then ensure it is of high quality and used with parents [54]. More screen use has been associated with increased dietary intake, poorer dietary quality, and overweight and obesity in young children [55]. Collectively, these feeding and nutrition challenges further impact young child nutrition.

*Knowledge and Surveillance Gaps*:

- A surveillance gap is documentation of various feeding challenges, particularly “picky” eating, and its impact on nutritional status, growth and development, health status, and physical activity level.
- There is a need for a consensus definition of “picky” eating and alignment of terminology.
- Greater dissemination of simple behavioral techniques to ameliorate feeding challenges will help caregivers work with their young children before needing formal clinical intervention and/or more serious disorders develop and are diagnosed.
- Additional research is needed to understand how external factors, particularly food insecurity and excessive screen media time, impact the feeding and nutrition of young children.

## 6. Conclusions

The first 1000 days provide an opportunity to optimize nutrition. Young children, particularly 12–24-month-olds, are an understudied population. Yet they are at risk for nutritional gaps, as they undergo rapid physical and cognitive growth and social development while transitioning to table foods and the family diet. Although the availability of food and nutrient guidelines are increasing and being updated for this age range, there are multiple surveillance and knowledge gaps to be filled both in the US and globally, notably prevalence and trends for sustained breastfeeding, education of healthcare professionals and caregivers on new recommendations for nutrient needs and dietary guidelines, collection of more dietary intake data especially for young child subpopulations, better standardization of the framework for YCFs/toddler drinks, defining/studying “picky” eating, and understanding the influence of external factors including those related to social determinants of health. Academia and clinicians, the private sector, and the government working together will help to close these knowledge and surveillance gaps both in the US and globally to help better support the nutrition and health of young children.

## Figures and Tables

**Table 1:** Compositional Guidelines and Expert Statements of Young Child Formulas (YCFs).

Author and Year	Population	Article Type	Main Topic	Age Range
Ladino et al., 2021 [30]	Latin American	Review	Considerations	0–36 months
Vandenplas et al., 2021 [31]	European	Review	Plant-Based Formulas	0–36 months
Verduci et al., 2021 [32]	European	Review	Clinical Studies	12–36 months
Hojsak et al., 2018 [29]	European	Systematic Review ^1^	Nutrient Composition	12–36 months
Suthutvoravut et al., 2015 [28]	International	Review ^2^	Composition Requirements	12–36 months
Vandenplas et al., 2014 [33]	Belgian	Review	Consensus Statement	12–36 months
EFSA 2013 [27]	European	Review ^3^	Nutrient Requirements	0–36 months
Przyrembel et al., 2013 [34]	European	Review	Rationale	12–36 months

^1^ European Society for Paediatric Gastroenterology, Hepatology, and Nutrition (ESPGHAN) Position Paper; ^2^ International Expert Group coordinated by the Nutrition Association of Thailand and the Early Nutrition Academy; ^3^ Scientific Opinion.

**Table 2:** Clinical Studies using Young Child Formulas (YCFs).

Author and Year	Population	Comparators	Duration	Serving Size/Day	Age Range
Rivera-Pasqual et al., 2020 [36]	Mexican	YCF with PUFAs vs. YCF without PUFAs	4 months	480 mL	12–30 months
Leung et al., 2020 [39]	Chinese	3 YCFs with bioactive proteins, 2′-FL HMO, and/or milk fat vs. Reference YCF	6 months	400 mL	12–30 months
Wall et al., 2019 [37], Lovell et al., 2018/2019/2021 [40,41,42,43]	Australian and New Zealand	GUM with reduced protein and energy vs. Unfortified cow’s milk	1 year	300 mL	12–23 months
Akkermans et al., 2017 [35]	German, Dutch, and English	YCF vs. Unfortified cow’s milk	20 weeks	≥150 mL	12–36 months
Xuan et al., 2013 [35]	Vietnamese	GUM with synbiotics and fortification vs. Control GUM	5 months	360 mL 5 days/week	18–36 months

## Data Availability

Not applicable.
